# Participation of communal cattle farmers in drought risk reduction in Southern Zimbabwe

**DOI:** 10.4102/jamba.v13i1.982

**Published:** 2021-05-14

**Authors:** Thabo Ndlovu, Johannes Belle, Mitulo Silengo

**Affiliations:** 1 Institute of Development Studies, Faculty of Commerce, National University of Science and Technology, Bulawayo, Zimbabwe; 2 Disaster Management Training and Education Centre, Faculty of Agriculture, University of the Free State, Bloemfontein, South Africa; 3 Disaster Management Centre, Mulungushi University, Kabwe, Zambia

**Keywords:** communal cattle farmers, drought risk reduction, have-not, participation preparedness

## Abstract

Communal cattle farming has remained the mainstay of many rural livelihoods in Zimbabwe and beyond. This was an enterprise that has stood the test of time, despite the increasing threats from drought shocks in the last two decades in Southern Africa. Prevalence of weather-related shocks was of concern, which had not galvanised communal farmers to actively engage in disaster risk reduction (DRR) initiatives in order to shield cattle from the negative effects of drought. In light of this development, this article examined the complexities of the involvement of communal farmers in DRR strategies to reduce the risk posed by drought on livestock in rural Umzingwane. This article used Arnstein’s Ladder of Participation to discern and generate insights on ways to promote the involvement of poor or vulnerable farmers or ‘have-nots’ in drought mitigation processes. This study adopted the descriptive survey design with 180 structured questionnaires administered to communal cattle farmers. Besides in-depth interviews, focus group discussions were held to examine the contributions of relevant stakeholders in driving the drought risk reduction agenda involving communal cattle farmers. This study revealed that limited investment options seriously affected farmers’ abilities to participate in drought risk reduction processes. Furthermore, farmers’ low-income levels and lack of well-defined drought risk reduction pathways did not offer the impetus to invest accordingly in drought mitigation. This article accentuated that successful drought risk reduction process were unachievable without the voice of the affected. Hence, development agencies should exceed placation and invest in strategies that propped philosophies of the vulnerable.

## Introduction

Community renewal has generated interest in disaster risk reduction (DRR), tilting the focus towards redistributing decision-making power as a key constituent for significant participation in humanitarian and development context. In one of the studies, De Vries (2016) corroborates that participation processes complement development interventions to entrench rights of vulnerable groups and deepen emancipatory processes. Participation facilitates sharing of experiences on DRR knowledge possessed by vulnerable and development practitioners (Alawadi & Dooling 2016) for improved decision-making. However, Thomas (2012) disqualifies power as a component of participation, arguing that its inclusion does little to aid the process. This made Arnstein’s (1969) Ladder of Participation model relevant in informing discussions in this article.

In contemporary drought risk management, marginalisation of the vulnerable groups is a thorny issue, making it inevitable to cushion the ‘have-nots’ against shocks, without engaging them. In Umzingwane district, livestock is a major source of livelihood and the area experiences high poverty-related deaths despite efforts to strengthen mitigation through deepening communal farmer participation in drought risk reduction interventions. Drought shocks continue to hurt the communal cattle farming community, thus challenging their coping, adaptation and livelihood options. Whilst the study area has central government, non-governmental organisations and local institutions with a mandate to champion the drought risk reduction discourse, the communal cattle farming community remains vulnerable to drought shocks. This exposes existing gaps in promoting the championing of context-specific drought risk interventions for the affected ‘have-nots’. To this end, this article sought to examine the role and participation of ‘have-nots’ in cattle drought risk reduction in Umzingwane. ‘Have-nots’ in this context entail drought-vulnerable farmers with limited space to advance their cattle management agenda, whilst communal farmer describes individuals practicing predominantly subsistence agriculture.

In the 1970s and 80s, participatory approaches were considered as the sine qua non of development that consequently led to people-centred actions (Penderis 2012). In concurrence with pro-poor development actions, Chambers (2008) suggests that the ‘have-nots’ deserve space to self-mobilise and consolidate their social capital if they are to develop networks that strengthen drought risk reduction efforts. Furthermore, Chambers (2008) affirms that properly conducted participation processes ultimately empower ‘have-nots’. As part of mobilisation, ‘have-nots’ in Zimbabwe have a history of coalescing through faith-based institutions, women and labour-sharing groups, as well as the more structured farmer organisations to attract relevant internal and external resources to address negative circumstances such as drought (Murisa 2011). As a consequence, participation helps ‘have-nots’ to realise some degree of transparency and accountability of livestock stakeholders, whilst accordingly, public processes legitimacy (ed. Cornwall 2011). This article discusses the theoretical framework underpinning the study, drought risk reduction and participation nexus as well as the methodology used to gather primary data and information. In response to whether communal cattle farmers participate in drought risk reduction, there are the results and discussion sections that culminate to the conclusions made in this article.

### Arnstein’s ladder of participation and disaster risk reduction

Disaster risk reduction applied to drought risk reduction in this article is inseparable from active involvement of the vulnerable, making Arnstein’s Ladder of Participation that comprises non-participation, tokenism and citizen power relevant. Employing Arnstein’s Ladder of Participation in this study helps us understand the important role that communities can play in reducing their vulnerability to impacts of climatic hazards that they may face. Through this ladder, the evolution and levels of participation of the disaster affected are interrogated in the context of communal cattle farming to pave the way for sustainable engagement. Non-participation constitutes the bottom rungs of the ladder, namely, manipulation and therapy typologies. Participation at this level entails playing a rubber-stamping role with no influence of the ‘have-nots’ on livestock drought risk reduction. This level of participation is in sharp contrast with tenets of DRR, which advocates for grassroots engagement and involvement. Non-participation restricts participants to selecting pre-developed solutions, a reflection of autocracy in the discharge of drought risk reduction strategies (Gonzalo-Turpin, Couix & Hazard 2008).

Tokenism, which is a notch above non-participation, constitutes informing, consulting and placation. The rungs present a form of participation with no assurance that ‘have-nots’ contributions would be embraced by decision makers (Mapuva 2014). Furthermore, tokenism describes a one-way and top-to-bottom information dissemination process with Mohammadi (2010:2) suggesting that it ‘is the illusion of being involved without the voice itself’. Placation involves mollifying, pacifying and appeasing farmers without due recognition of their contribution in drought risk reduction (Mapuva 2014). In other words, non-participation and tokenism reflect suppression from voicing on the best trajectory for sustained drought risk reduction. Stifling participation of farmers perpetuates a situation whereby power holders determine the outcomes of the proposed DRR interventions whilst upholding legitimacy by referring to rhetorical farmers’ inclusion (Zocher 2010). Finally, citizen power, which is the top-level rung, involves partnership, delegated power and citizen ownership and control of DRR measures, which ultimately links the vulnerable with development agencies in a mutual beneficial manner. The work of Cooke and Kothari (eds. 2001) concurs that participation should aim to make have-nots central to their development by fostering beneficial engagements in interventions that affect them.

Through citizen power, transformational processes that foster shared decision-making in planning, implementation and evaluation of DRR interventions are envisaged to prevail (King & Zanetti 2005). Tritter and McCallum (2006) dispute Arnstein’s view that participation is hierarchical in nature with community control being the ultimate goal, by suggesting that control is not the ultimate aim given different reasons for involvement in decision-making.

## Drought risk reduction and communal farmer participation

Drought is a creeping natural hazard, characterised by extended periods of limited precipitation (Sivakumar et al. 2010), and has huge consequences on humans, livestock and natural grazing resources quantity and quality (Garbero & Muttarak 2013). The creeping nature of the hazard allows communities time to devise counter strategies and invest in drought risk reduction (Habiba, Shaw & Takeuchi 2011). Drought is one of the major hazards that affect the livelihoods of the community in Umzingwane. The involvement of communal farmers in DRR activities, not as passive participants but as active participants, is cardinal in reducing their vulnerability and promoting resilient livelihoods. This study focuses on meteorological drought that is commonly described by the degree of departure of precipitation from the average normal. Its impacts are disproportionately spread because of vulnerability profiles in communal farming areas with notable setbacks in livestock development and attainment of household food security (Sivakumar et al. 2014). Unfortunately, the drought affected communities and the DRR practitioners always suffer from a hydro-illogical cycle, whereby previous drought experiences are not used to inform proactive future planning for the next drought (Belle 2016). Weather-related effects are likely to last given that the African continent is predicted to experience extreme and widespread drought impacts as a result of slow drought risk reduction progress, increasing population, reliance on rain-fed agriculture and the degradation of the environment (Masih et al. 2014).

International Federation of Red Cross and Red Crescent Societies (IFRC 2012) explains drought risk reduction as systematic efforts to analyse and manage the causal factors of droughts including lessening exposure to the phenomenon, vulnerability and wise management of land to enhance preparedness for its adverse effects. Disaster risk reduction measures normally begin with risk assessment comprising hazard, vulnerability and capacity assessments (Mercer 2010), all of which require inputs from the ‘have-nots’. Disaster risk reduction has become fashionable (Alexander 2013) although with less benefit to societies directly impacted by disasters (UNISDR 2009). Disaster risk reduction is multi-disciplinary in nature (Wisner et al. 2004) and focuses on broader social, political, environmental and economic context of the stressor (Weichselgartner & Obersteiner 2002). Disaster risk reduction strategies relevant to the cattle industry include establishment of fodder banks, grazing control, early warning systems and risk transfer. However, these measures are seldom practiced in rural Zimbabwe. As corroborated by Vetter (2013), destocking is rarely implemented as most farmers do not have cattle or have less than what they need. The 2019 Zimbabwe Vulnerability Assessment Committee (ZIMVAC) confirmed that 57% of rural households in Matabeleland South Province, under which Umzingwane district falls, did not own cattle.

Blending grassroots initiatives with relevant top-down strategies becomes inescapable in DRR (Mercer 2010; Wisner, Gaillard & Kelman 2012). To heighten local action, international frameworks for DRR, namely, the Yokohama Strategy and Plan of Action for a Safer World, the Hyogo Framework for Action 2005–2015 and the Sendai Framework for DRR 2015–2030 (SFDRR), all demonstrate the essence of community actors (Poterie & Baudoin 2015; UNISDR 2015). Whereas the Hyogo Framework for Action 2005–2015 was complex with minimal development benefits (O’Faircheallaigh 2010), the SFDRR prioritises the understanding of risk and investment in resilience building through engaging the concerned players. Of relevance to this article, is the SFDRR priority number four that emphasises the strengthening of preparedness to shocks through deepening participatory processes (UNISDR 2017).

Another international framework with DRR connotations is the United Nations Framework Convention on Climate Change; Conference of Parties (COP21), which in 2015 compelled action on climate change mitigation and climate change adaptation (CCA). Whilst there are many points of convergence between DRR and CCA, the major difference is that DRR seeks to lessen vulnerability to all hazards, whilst CCA focuses on climate related hazards (Mercer 2010). Whereas international policy frameworks are welcomed, Mercer et al. (2012) argue that they are non-binding treaties with no tangible targets and that they remain unclear to entail concrete outcomes at the national level. Participation of local actors and communities is central in the aforementioned DRR frameworks to deepen resilience, tackle socio-economic vulnerabilities and not inadvertently increase or create new risks (Twigg 2015). This trend strengthens the realisation that local knowledge facilitates the processes of DRR in a cost-effective, participatory and sustainable manner (Howell 2003).

This article explores communal farmer participation in drought risk reduction. This participation entails an inclusive process in which all key players are involved in drought mitigation activities and, more importantly, have some degree of influence over decisions that affect them (Arnstein 1969; Robinson et al. 2010; Sinclair & Diduck 2009). Mansuri and Rao (2012) deem participation as a reaction against ‘top down’ approaches that offer ‘have-nots’ limited opportunities to influence decisions. Interestingly, command-and-control and top-down methodologies continue to guide DRR processes with more emphasis on scientific ideas and national government decisions at the expense of grassroots participation and actions (IFRC 2012). Whilst some scholars view top-down and bottom-up approaches negatively, Mercer et al. (2012) see them as complementary, valuable and compulsory to sustain DRR efforts. Investing in participation proffers a paradigm shift through strengthening the association between the power holders (‘uppers’) and local vulnerable people (‘lowers’) (Chambers 1994; Williams 2004). It is critical to realise that DRR interventions based exclusively on solid scientific parameters fail to conform to the local context, including those decisions informed by local knowledge only because of evolving environments, as this creates the need to combine applicable local and scientific knowledge (Mercer et al. 2012).

The theory of participation put to test the notion that ‘everyone has a right to be involved in the cultural life of the community’ (Bollo et al. 2012:7), as espoused in Article 27 of the United Nations Universal Declaration of Human Rights (Jancovich 2015). The declaration does not give leverage to the educated and wealthy to influence DRR processes. It accentuates inclusion of all for the collective scrutiny of proposed drought risk reduction interventions and lessens the deleterious unchallenged assumptions (Burton & Mustelin 2013; Fischer 2003). Therefore, subjecting an intervention to debate improves manageability and eliminates conflicts. Robinson et al. (2010) profess that collective decisions are not always premised on well-reasoned exchange of ideas but may exclude other players for convenience. Race, ethnicity, health, education, income levels, emanating from the scarcity of livelihood options for the poor, are valuable determinants of vulnerability (Adger, Brooks & Kelly 2004; Cardona 2011). Consequently, communal households that attain higher educational levels are considered more tolerant to drought stress because of their propensity for increased adaptive capacity (Garbero & Muttarak 2013). Drought risk reduction success thrives on information that educated societies are likely to access easier than the least schooled (Jerit, Barabas & Bolsen 2006). Participation is either active or passive. Passive participation entails involvement of farmers through one-way information sharing with no assurance that they will influence outcomes of DRR planning (Boyer-Villemaire et al. 2014). Such engagement limits the voice, inclusion and commitment to the entire drought risk reduction process. Active participation refers to two-way exchanges through decisional forums, making involvement inescapable for the aged and informed populations (Hedjazi & Arabi 2009). This is reflected in that communities become informed of their circumstances; they set standards of addressing drought-related matter (Boyer-Villemaire et al. 2014). Wealth offers disproportionate propensity to participate in DRR whereby the more vulnerable engage less and have less ‘voice’ (Alesina & La Ferrana 2000). Knutson et al. (2011) and Pica-Ciamarra et al. (2011) corroborate that the low-income infringes in their ability to participate in DRR. Such conditions deepen the wait-and-see attitude in ‘have-nots’ as they anticipate external support and hope that the situation will improve. Impediments to effective engagement draw from weak traditional institutions, poor rural infrastructure and limited technical assistance that compounds mobilisation of ‘have-nots’ for collective decision-making (Natawidjaja et al. 2014). The articles contribute to the policy discussions on community participation in DRR as well as practices of engaging vulnerable groups such as switching to more drought-tolerant breeds, which appeared impossible as a DRR intervention, owing to high replacement costs and non-availability of preferred breeds (Clarke, Shackleton & Powell 2012). It is therefore important to bring together these pros and cons on grassroots involvement and participation in the context of drought risk reduction for communal cattle farmers.

## Study area

Umzingwane district, which lies at the north-eastern side of Matabeleland South Province, shares its boundaries with Gwanda to the east, Umguza to the north and Bulawayo to the west. The district falls under agroecological zone (AEZ) IV and V. The area receives up to 450 mm of rainfall annually for wards in AEZ IV and less than 400 mm for AEZ V. The dominant landforms are bare granite hills and hills covered with vegetation, separated by areas of flat lands and occasional flat rock structures. Livestock and crop farming, vending, artisanal mining and remittances are key livelihood sources for the Umzingwane community. What should be borne in mind is that tilling the land is one of the important activities that communal farmers embark on to enhance household food security, for which cattle provides manure and draft power. The prominence of livestock as a source of livelihood is rooted in the local culture, which deems households without this asset as poor.

## Methodology

The research, which is underpinned by Arnstein’s theory of participation, was largely qualitative. Arnstein’s Ladder of Participation, which comprises non-participation, tokenism and citizen power, was employed to aid the study respond to the question on how vulnerable communal livestock farmers are involved in drought risk reduction in Umzingwane district. Using this ladder, the evolution and levels of participation of the disaster affected are interrogated in the context of communal cattle farming as a way for sustainable engagement. The quest to influence liberalisation of marginalised communal farmers from unjust processes inspired the use of the qualitative approach to do in-depth discussion so as to magnify voices of vulnerable groups for effective change. Umzingwane district was selected because of its high reliance on livestock as a livelihood and because of poverty-related deaths experienced by this community.

The study was conducted between August and December 2019 following granting of permission by the Provincial and District Offices of the Ministry of Local Government and National Housing to account for farmer’s priorities and perceptions on participation in DRR. The traditional leadership was engaged and they facilitated meetings with respondents and other key informants within their jurisdiction.

A descriptive survey approach informed the collection of relevant data and it provided a deep analysis on the influence of education and income levels on farmer participation in communal livestock farming areas. The research design offered flexibility on the choice of data collection instruments that were administered by local Agriculture Technical and Extension Services (AGRITEX) officers and enumerators. The study exploited the relationship that existed between local extension personnel and communal farmers to administer structured instruments, which focused largely on demographics, livestock ownership, number of cattle sold, income levels and cattle losses.

Data were collected using structured questionnaire, in-depth interviews and focus group discussions. Structured questions focused largely on age and gender of stockowners, income levels and education; cattle lost and sold because of drought. In-depth studies gathered data engagement preferences by respondents and challenges faced by communal farmers and various institutions in effectively participating in drought mitigation. The targeting of research participants was guided by simple random and purposive sampling techniques. Simple random sampling offered every respondent an equal chance of participating in the study. To this end, a sample size of 180 communal cattle owners was drawn from six dip tanks with a total population of 3324 stock owners using simple random sampling techniques, translating to an average of 30 respondents per dip tank. The ward cattle list guided targeting, with every communal cattle owner assigned a number and thrown into a hat from which a representative sample was randomly picked. Purposive selection, which largely focused on those knowledgeable about the subject matter, targeted key informant participants for in-depth and focus group discussion processes to generate insights into drought risk reduction engagement in the area. The purposively targeted individuals were representatives from the AGRITEX, Veterinary Services, Emergency Management Agency, local chiefs and village heads.

Diversity of characters was facilitated by judgmental sampling whose extremes were considered based on one’s role in livestock production, position in society and other background attributes such as present and past cattle ownership. Unstructured data were gathered by the authors targeting key informants with appointments made through the local leadership for discussions with livestock owners and Livestock Development Committees (LDC) at sites considered convenient to respondents. Themes on engagement preferences and challenges faced by farmers guided the processing of data generated by in-depth and focus group processes, whilst structured data on gender, age, educational and income levels were analysed using the Statistical Package for Social Science (SPSS). The analysis followed both inductive and deductive thematic analysis approaches. Using SPSS version 23, ownership trends, sales and losses because of drought were processed and presented in tables, graphs and pie charts.

## Results and discussion

This section focuses on gender and age of respondents as well as education, cattle sales and loss incurred by communal farmers, engagement preferences and challenges encountered in participating in drought risk reduction.

### Age of respondents

**FIGURE 1:**
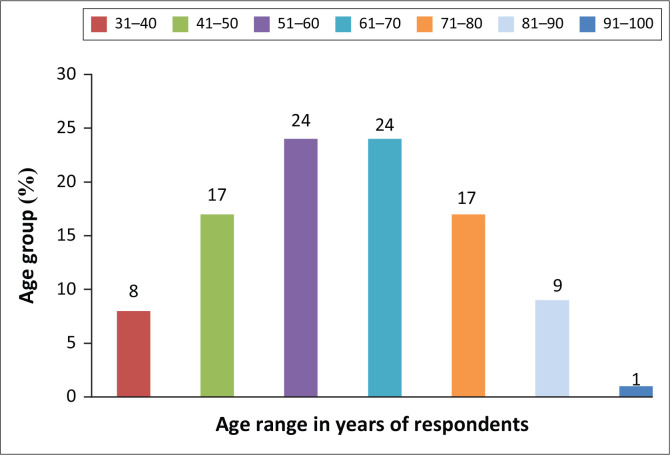
Age-specific distribution of respondents.

Age is one of the important factors that define ownership and management of livestock in communal setting. Figure 1 details the age distribution of respondents and the implication for decision-making and DRR.

Figure 1 depicts the age distribution of respondents in years, with the majority falling within the 51–60 (24%) and 61–70 (24%) age groups. Those aged 41–50 and 71–80 both accounted for 17%, with the 81–90 age group recording 9%, whilst the 31–40 years age group recorded 8%, with 91–100 being the least at 1%. The 81–100 age group was narrowly represented as only a few respondents normally reach that stage. The 51–70 age group appears to be the prime age of owning cattle and hence, their participation in DRR is likely to be more to preserve livestock, which forms the backbone for survival in rural settings as the majority from this group could be out of employment. The 41–50 group signifies the age at which farmers start to build their cattle herd and subsequently decide on DRR strategies to adopt, whilst the 81–90 age group reflects a constituency that relies heavily on previous experiences and indigenous knowledge systems and may at times be impervious to scientific DRR strategies.

Figure 1 shows that cattle ownership rarely starts at a younger age. This affirms that cattle production is not an activity that identifies well with younger generations as they are mobile and seeking lucrative ventures to establish and anchor their future. The younger generation does provide assistance to families in looking after their livestock although with minimal influence on decision-making. Mudzielwana (2015:73) concur that ‘the likelihood of owning livestock increased with the age of the household head,’ a reflection that the participation of younger generation in DRR with regard to livestock and drought is less. The absence of younger generations robs the livestock sector of innovative ideas to transform disaster risk management.

### Education

Drought risk reduction implementation success and failure hinge on the examination of the context and hence, analysis of the education, cattle sales and income levels was analysed to discern how they could influence prevailing behaviours and attitudes when faced with shocks. Table 1 shows community wide influence of education and income levels on DRR in Umzingwane district.

**TABLE 1:** Education, cattle sales, losses and income levels for cattle owners.

Activity	Never went to school	Primary	Secondary	Vocational trained	Tertiary
Education (%)	3	53	39	3	2
Cattle ownership or farmer	2.8	4.1	5.2	7.4	14.6
Cattle sold per farmer	0.33	0.95	0.92	0.80	0.00
Cattle lost per farmer	1.00	0.98	0.94	0.00	1.3
Sales of cattle owned (%)	12	23	24	11	0
Income level per day ($)	0.25	0.35	0.50	2.80	11.00

The majority of respondents reported primary (53%) and secondary (39%) education as their highest levels of study with the ‘never went to school’ (3%), with vocational and tertiary levels (2%) being the least. Whilst the majority of farmers are literate, having higher number of primary school trained respondents could impede transfer of highly scientific DRR activities and subsequently constrain adoption of such interventions. In corroboration with the biblical saying ‘my people perish because of lack of vision’ (Proverbs 29:18), of note is that drought risk reduction demands formulation of short- and long-term strategies, which may become difficult for the less informed and educated communities. The issue of illiteracy becomes prominent given contemporary bias towards scientific drought management measures at the expense of indigenous knowledge strategies, which gives the more literate an advantage to explore available options.

Vocational and tertiary trained respondents were few, making the need to promote and support attainment of levels such as very relevant as education helps build confidence to tackle vulnerable circumstances such that those deemed illiterate may lack the necessary skills for effective engagement. One of the village heads said, ‘This is for the educated’, suggesting that only the literate group should be engaged in DRR. What compounds matter for the less educated is the limited practice of indigenous systems, which are well understood and are maintenance free from resource-constrained settings. Anaeto et al. (2012) concur that the ability of farmers to respond positively to new ideas is premised on how educated they are to apply them. Boyer-Villemaire et al. (2014) aver that involvement in local development is unavoidable in highly educated society.

### Income level

The study shows that as income levels increase with level of respondent’s level of education, so does cattle ownership. Whilst cattle lost because of drought decreases with the level of respondents’ education, the result is contrary with the tertiary trained respondents. This is in sharp contrast to the findings of Garbero and Muttarak’s (2013) that communal households that attain higher educational levels are deemed more tolerant to drought stress because of their propensity of increased adaptive capacity.

The results also indicate that as cattle loses because of drought decrease, income increases from $0.25/day category up to $2.80/day. The results resonate with Sun et al. (2012) that increase in income levels of households, strengthened resilience, and this position changes with the respondents in the high-income level ($11.00). The representation of the high-income respondents was insignificant for the study to be conclusive about this result. The trend from the $0.25/day category up to $2.80/day indicates the capacity to infuse drought risk reduction strategies such as supplementary feeding in their management calendar. Income gradations reflect challenges faced in accessing capital to bankroll drought risk reduction interventions in communal areas with some failing to raise the required collateral. Respondents lost more cattle than they sold, except for that vocationally trained whose results indicate that they sold more than what they lost.

The desire to accumulate more cattle and limited investment options in communal areas deepens the wait-and-see attitude amongst farmers despite early warnings and imminence of threats, which often contributes to massive cattle deterioration. The prestige and status bestowed by virtue of owning cattle result in huge losses as communal farmers seek to preserve their standing by keeping as much cattle as they can, thereby perpetuating vulnerable cattle-rearing conditions. The lack of confidence in the banking sector in Zimbabwe partly accounts for attitudes displayed by farmers with most of them holding onto cattle as the best store of value. What should be borne in mind is that tilling the land is one of the important activities that communal farmers embark on to enhance household food security where cattle provides manure and draft power. It is clear that communal farmers have a plethora of needs that are social, economic and cultural; all of which depend on cattle and hence, they thrive to keep more to meet diverse needs. Cattle sales are the last resort for most communal farmers. Cattle markets cannot be taken lightly as they force communal farmers to withhold their stock hoping for the best outcomes. Although communal farmers professed scantiness of information on markets, unclear restocking pathways and costs debar implementation of drought risk strategies.

Chisango, Tembachako and Mupoperi (2015) and Jordaan et al. (2017) aver that unfair prices from private buyers and scarce financial capital to restock make it extremely difficult for communal farmers to participate in DRR through destocking. The findings corroborate Rootman, Stevens and Mollel (2015) study in Sekhukhune District that livestock owners generate little income from cattle. This reflects a common trend that communal farmers generate little financial contribution from cattle to support their livelihoods.

## Farmer engagement preferences

**FIGURE 2:**
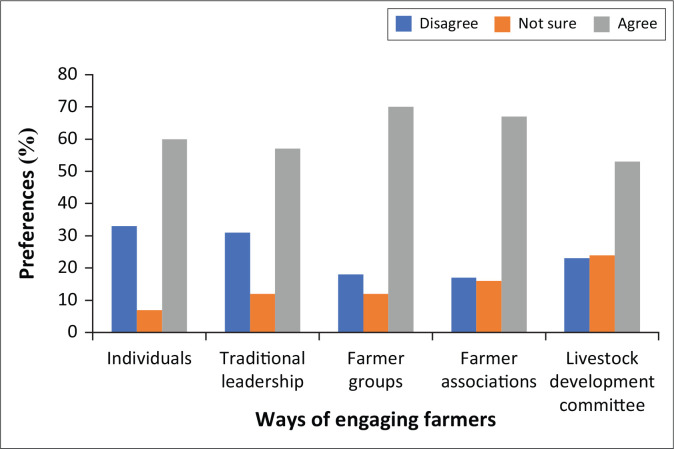
Farmer engagement preferences.

The role of institutions in DRR cannot be down played together with the approaches in engaging the vulnerable groups to encourage adoption of recommended strategies. The mode of engagement has ramifications on the level of engagement and the DRR strategies adopted. To this end, it is important for institutions to select context-specific engagement methodologies to enhance participation of vulnerable groups in DRR. Figure 2 shows respondents’ engagement preferences.

Respondents preferred to be engaged as groups to fully embrace cattle drought risk reduction initiatives. Group engagement builds synergies and promotes cohesion within the farming community. Positive relationships amongst communities become a special ingredient for successful learning and adoption of relevant answers to drought. The finding concurs with Wellard (2013) that working with farmers in groups is a famous approach because of its relevance in generating common interest and reaching out to many farmers in a cost-effective manner. Working in groups is not a new concept as history informs traces of communities pulling together through what is locally called ‘ilima’ where a farmer invites colleagues for a day or two for support on a defined task. In the process, the organiser prepares meals for the supportive group and this creates bonds that spread beyond a single activity. Despite various perceptions, group engagement easily sways attitudes towards group agendas for enhanced complementarity and it curtails resource mobilisation hiccups. Although group engagement develops synergies, the dynamics of keeping them together diminishes group efforts. Additionally, engaging ‘have-nots’ becomes tyrannical if group dynamics entrenches desires of the uppers at the expense of the marginalised and vulnerable members.

Farmers’ associations were very popular with respondents as engagement avenues because of the ease with which they could lobby government and other development agencies to respond to farmers’ concerns. This result confirms farmer organisations such as Zimbabwe Farmers Union (ZFU) that were and are still in existence to help deal with challenges bewildering communal farming areas through negotiations and advocacy and to advance their interests. In-depth discussions bemoaned the docility of these organisations in championing the livestock drought risk reduction agenda. Farmers cited exorbitant annual subscription fee as a huge impediment to the existence and functioning of farmer associations to fulfil their mandate. Respondents were ignorant of the fact that ZFU drew most of its financial resources from the sale of membership cards and levies, with government providing minimal support. The study found out that in the post-independence era, farmers benefitted from restocking exercises, drugs and other livestock inputs from ZFU, although without mention of its influence on grazing conditions. Individual engagements were singled by respondents as important and necessary between stock owners and technical institutions. Results further indicate that one on one encounters with respondents garnered much attention by the extension personnel and solutions derived were unique to the individual, as compared to group engagements. The challenge with this method is the extension officers: farmers’ ratio that results in poor turnaround times. However, it is critical to note that farmers’ choices and capabilities are easily exploited if engaged individually than when performed in groups, a view shared by Cooke and Kothari (eds. 2001) that the presence of an external facilitator sways decision away from the ‘lowers’, given the significant power the ‘uppers’ possess. Individual engagement, whilst presenting opportunities for balanced outcomes, grants external personnel more strength by virtue of being an officer.

Traditional leaders are an appreciated and important constituency in drought risk reduction, with huge significance for sustenance. To affirm this culture, one of the farmers said ‘we submit to our leadership’, implying that involving traditional structures in drought mitigation projects builds community trust and facilitates monitoring against compliance with behavioural norms of the area. Responses against the traditional leadership role in DRR emanated from discontent with the way they handled the targeting processes of food-insecure households. Ballard (2016) asserts that the challenge with engagement via traditional institutions is that they can paradoxically serve to demobilise rather than mobilising communities. Demobilisation is guaranteed if local interests are shunned in decision-making, forcing some to withdraw their commitment. The traditional institution setback is that they deal with a wide range of development processes, some of which they lack the capacity to handle. The complexity of some DRR procedures frustrates ‘have-nots’ leading to the majority tiring and abandoning their contribution. Livestock development committees (LDC) are an avenue through which communal farmers are engaged, although some cast aspersion on their effectiveness in this role. Respondents recognised the LDC for its active participation in the management of dip-tank infrastructure and support to animal health initiatives such as vaccinations and dipping of cattle. The LDC was said to profoundly provide a communication link between the department of Veterinary Services and the entire cattle farming community in ensuring uninterrupted dipping schedules. To enhance preparedness, the LDC advises communal farmers on vaccination schedules and plays a significant role in ensuring that society is ready for droughts and other threats to the livestock sector. Of concern is their limited scope as they focus solely on animal health issues, ignoring production matter such as grazing. The engagement between farmers and government institutions was weak despite the fact that stock owners are open to engagement as individuals, farmer groups, farmer associations and LDC, some of which are cost-effective and could be explored. One of the LDC chairpersons said, ‘We value knowledge from all spheres’: a reflection of openness to all development agencies. Essentially, partnerships as enshrined in Arnstein’s theory on participation would make it easier for community interests to be harnessed through preferred approaches of engagements for effective programming on DRR. Nonetheless, engaging farmers in groups, through farmer associations and individually, each with its own merit, were deemed the most convenient in encouraging participation of communal farmers on drought matters.

## Drought risk reduction participation challenges

**FIGURE 3:**
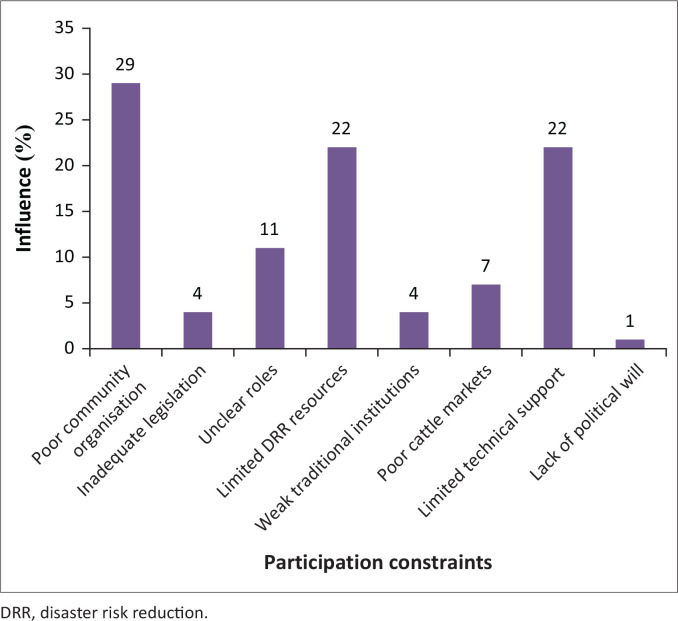
Communal farmer participation constraints.

Participation of communal cattle farmers in drought risk reduction faces a plethora of internal and external challenges. Internal issues are mostly within the farmers’ capacities to handle, whilst external threats present forces too heavy to contain by communal livestock players. Figure 3 details factors limiting farmers’ participation in drought risk reduction in the study area.

Poor community organisation, a reflection of lack of common agenda, lack of cohesion and weak mobilisation initiatives were the major drivers of farmers’ failure to engage in drought risk reduction.

Communal farmers’ vulnerability to low drought thresholds signifies the level of disjointedness in creating synergies and containing shocks within their capacity. Failure to relate individual interests to community agenda contributes to a disorganised society with no itinerary to deal with glaring challenges. Local conflicts entrench division, suspicion and ultimately blur local vision, which further deepen susceptibility to drought conditions. Disorganisation debars effective community resource deployment by giving the more powerful members space to exploit at the expense of collective efforts. The lack of self-organisation to steer cattle development projects without external influence is compounded by the dearth in knowledge on drought preparedness.

It is in this context that extension agents need to be exposed to the basics of community organisation and group management skills to understand the impediments to collective efforts within communal sectors. Owos-Oab (2014) and Pascucci et al. (2013) corroborate that poor and weaker communal farmers need assistance to organise themselves for effective drought mitigation as the failure to self-mobilise, create chaos and impair roll-out of drought risk reduction initiatives. The limited drought risk reduction material, financial resources and technical support render cattle farmers too weak to deal with livestock during drought.

Lack of clarity of roles between communal farmers, government and non-governmental organisations (NGOs) creates confusion in initiating cattle drought mitigation programmes. Confusion of roles emanate from the absence of a full-fledged DRR unit that sets the agenda and galvanises the society. The traditional leadership roles in common property appear cloudy and hence, no one is held accountable for veld degradation. The issue of roles has caused communal farmers to believe that government institutions must at all times direct them if they are to act despite the fact that they have a duty to protect their animals from shocks. Interactions with key informants suggested that farmers are aware of what is expected of them; however, they wait until it is too late to implement relevant DRR measures as some believe that the cattle can withstand drought conditions. The poor rural infrastructure and limited technical assistance compound the challenge of working in groups (Natawidjaja et al. 2014). According to Aref (2010), weak government institutions deter participation of communal farmers in DRR because of poor knowledge transfer, which contributes to discontinuity of interventions (Ladele & Ayoola 2011).

The notion that farmers lacked technical support was disputed by extension officers as they argued that communal farmers are capable but fail to work on their own without external influence. In-depth discussions with extension officers and LDC representatives showed that donor syndrome was crippling the successful design and implementation of cattle drought adaptation initiatives. One of the Livestock Production Development officers said, ‘Subsistence mind is in control amongst cattle owners’ (November 2019), insinuating that communal farmers disregard their potential and rely heavily on external support for drought solutions. Furthermore, farmers do not treat cattle production as a business but rather continue to make uneconomic decisions, which have no bearing on the growth of the beef industry.

## Conclusion

This article makes three major findings: firstly, on farmer’s income levels and DRR participation nexus, next on the implications of mode of engagement and finally on poor community organisation on DRR processes by communal cattle farmers in Umzingwane. Whilst education does offer the knowledge and skills to absorb drought shocks and instil a culture of safety, the findings reveal that the majority of communities with basic education are low-income earners who struggle to propagate the DRR concept in an environment with limited investment options. The low-income level makes it difficult for farmers to invest in drought mitigation and hence, this is contributing to the confinement of DRR to non-participation and tokenism, where farmers rely heavily on pre-determined strategies from power-holders. To this end, formulation of compatible drought risk reduction strategies is unachievable without the voice of the affected using their preferred mode of engagement and hence, development agencies should exceed placation and invest in strategies that prop philosophies of the vulnerable. This can be achieved when institutions exercise flexibility in their DRR approaches to facilitate adaptation and coping capacities to the ever-changing frequencies and magnitudes of drought.
